# Crown-Root Fracture Treated With Super-Bond—A Case Report With 55 Months Clinical Follow-Up

**DOI:** 10.1155/crid/6104187

**Published:** 2025-07-08

**Authors:** Yongwei Li, Hanlin Deng, Chan Lu, Shujie Li, Jiaqin Tang, Linyin Huang, Yingjiao Nong, Lihua Liang

**Affiliations:** Department of Oral Implantology, Affiliated Stomatology Hospital of Guilin Medical University, Guilin, China

**Keywords:** biological width, conservative treatment, crown reattachment, crown-root fracture, minimally invasive

## Abstract

Crown-root fragment reattachment is an efficient method for restoring fractured teeth but is prone to failure due to complications such as microleakage or improper alignment, particularly when moisture control is compromised. In this case, the maxillary left central incisor that had sustained a complicated crown-root fracture was successfully reattached through a meticulously planned adhesive protocol. Specifically, precise visual reattachment on a small periodontal flap using the Super-Bond adhesive system in a controlled moisture led to favorable long-term results. The 55-month follow-up shows that conservative reattachment can restore function and appearance and preserve periodontal health.

## 1. Introduction

With increasing participation in outdoor sports and higher traffic density, the incidence of dental trauma has escalated, particularly affecting the anterior teeth in the esthetic zone [1, 2]. Epidemiological studies indicate that dental trauma most commonly affects children aged 6–10 years, particularly males, as they are more frequently engaged in physical activities involving contact (male-to-female ratio of 2.5:1), with crown-root fractures accounting for approximately 5% of all dental injuries [3]. When the fracture involves the pulp, tooth discoloration (such as yellow or gray) may occur, significantly affecting dentition, overall esthetic outcomes, and the occlusal relationship [4]. Timely diagnosis and further therapeutic intervention are imperative to prevent potential esthetic deficits and psychological sequelae [5].

The conventional treatment options for crown-root fracture involving the biological width include crown lengthening, orthodontic extrusion, immediate implant placement, or postextraction implant–supported prosthesis restoration [6]. However, these treatments are often time-consuming, nonconservative, and involve greater invasion of teeth or bone tissue, as well as an uncertain prognosis [3, 6]. With advances in adhesive technology and material properties, reattachment of fractured teeth has received more attention as a viable alternative, which is a conservative, less invasive, and economical treatment, first described by Rathod et al. [7]. According to the International Association of Dental Trauma 2020 guidelines, reattachment of the fractured tooth fragment is defined as a treatment for crown-root fractures [8].

However, as crown-root fractures tend to result in compromised biological widths of teeth, accurate fragment alignment and moisture control are crucial for successful reattachment. The Super-Bond adhesive system, a widely used resin-based adhesive, has shown excellent bonding strength and durability due to its unique 4-methacryloxyethyl trimellitate anhydride/methyl methacrylate-tri-n-butyl borane resin composition, which enhances adhesion to both enamel and dentin while providing superior moisture tolerance [9]. Hence, this paper describes a case of immediate reattachment and restoration performed under a small periodontal flap following a modified minimally invasive surgical technique (M-MIST), utilizing strict moisture isolation and the Super-Bond adhesive system, which ultimately achieved favorable clinical efficacy [10].

## 2. Case Report

### 2.1. Diagnosis and Etiology

**Figure 1:**
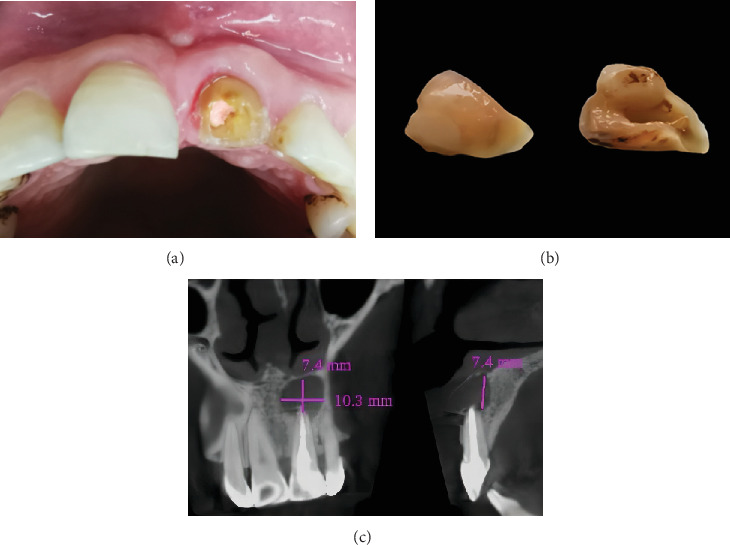
Preoperative examination. (a) Intraoral examination: oblique fracture extending from the palatal to the labial. (b) View of the fractured segment. (c) CBCT reconstruction (before fracture): 7 × 10 mm elliptical low-density shadow was observed in the apical region.

In 2020, a 54-year-old female patient presented to our hospital within 1 h following a fractured maxillary left central incisor, which had been stored in water prior to arrival. The patient reported no medical history and systemic diseases, and her family history was unremarkable. The tooth had undergone root canal treatment and cyst removal surgery a year ago, without subsequent crown protection. Clinical examination revealed the discolored tooth with an oblique fracture from the palatal to the labial, extending approximately 3 mm from the gingival margin (Figure 1a). The fractured segment had fallen out (Figure 1b). The x-ray examination results are shown in Figure 1c. The diagnosis was an oblique crown-root fracture of the maxillary left central incisor.

### 2.2. Treatment Alternatives

Several treatment options, including their advantages and disadvantages, the associated cost, and prognosis, were explained to the patient. The patient expressed a strong desire to preserve the natural tooth and opted for reattachment with fiber post reinforcement.

### 2.3. Treatment Objectives

The primary treatment objectives for this case were divided into three phases, adhering to conservative and minimally invasive principles while addressing the patient's strong desire to preserve her natural dentition (Table 1).

The first phase focused on the immediate reattachment of the fractured crown-root segment using advanced adhesive techniques to restore both function and esthetics. Emphasis was placed on ensuring precise fragment alignment, strict moisture control, and the use of the Super-Bond adhesive system to achieve a durable and stable restoration. This phase was aimed at minimizing the risk of microleakage or debonding while promoting biological width remodeling and avoiding any compromise to periodontal health. Reinforcement of the fractured tooth with a fiber post was also performed to enhance structural integrity and prevent future fractures.

Following successful reattachment and confirmation of favorable healing outcomes during follow-up, the second phase involved the placement of a full-ceramic crown to provide long-term protection and esthetic integration with the surrounding dentition.

The final treatment phase incorporated comprehensive long-term monitoring through scheduled clinical and radiographic examinations to evaluate prosthetic integrity, functional occlusion, periodontal health status, periapical healing progression, and alveolar bone remodeling dynamics, ensuring the sustained success of therapeutic outcomes.

### 2.4. Treatment Progress

The fractured crown was disinfected with a 2% chlorhexidine solution (Chlorhexidine Gluconate Solution, Longly Biotechnology, Wuhan, China) and then meticulously stored in 0.9% physiological saline to prevent dehydration and preserve its bonding viability [11]. The post space was prepared on the lingual aspect using diamond burs (Dia-Burs, Mani, Tochigi, Japan) to merely remove obstructing tooth structure and establish a straight-line pathway for the #1.4 fiber post, ensuring passive seating into the root canal (Figure 1b). Meanwhile, the post space within the root canal was prepared and shaped with Peeso reamers (Peeso Reamers, Mani, Tochigi, Japan).

**Figure 2:**
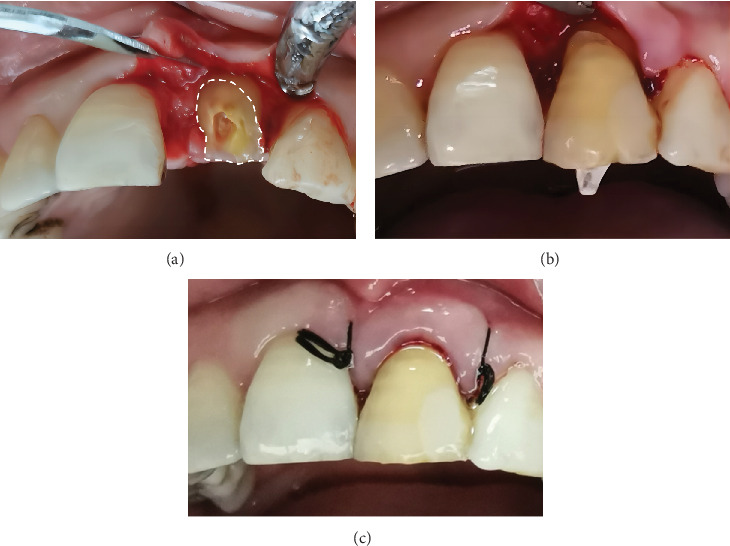
Fragment reattachment and flap suture. (a) Following the elevation of the labial mucoperiosteal flap on the affected tooth, the fracture line was observed clearly. (b) Fragment reattachment reinforced with fiber post. (c) Sutured flap.

Then, following the M-MIST incision design, a small, full-thickness labial mucoperiosteal flap was elevated to clearly expose the root surface fracture line on the affected tooth, while the lingual flap remained unreflected to preserve the interdental gingival papilla (Figure 2a) [10]. This approach ensured optimal visibility for accurate alignment during fragment reattachment.

To control moisture, two saliva ejectors were strategically placed palatally to remove blood from the mesial and distal interdental spaces, preventing contamination during bonding. Gingival hemostatic gel containing ferric sulfate (ViscoStat Clear, Ultradent, South Jordan, United States) was applied locally to control bleeding and maintain a dry field for the bonding procedure [12].

Next, the fractured segment was etched with 37% phosphoric acid (Scotchbond Universal Etchant; 3M ESPE, St. Paul, United States), and the root surface was coated with a dentin conditioning agent (Super-Bond C&B, Nissin Dental Products, Kyoto, Japan) to enhance dentin bonding strength [13]. Then, the two parts were rinsed and dried in preparation for bonding.

Finally, the fractured segment, the #1.4 fiber post (Matchpost, RTD, Saint-Egrève, France), and root surface were coated with Super-Bond cement (Super-Bond C&B, Nissin Dental Products, Kyoto, Japan) [9]. The fractured segment was reattached using the fiber post to reinforce the bond between the fragment and the root (Figure 2b) [14]. Most importantly, special attention was given to visually align the fragment accurately during the reattachment process.

**Figure 3:**
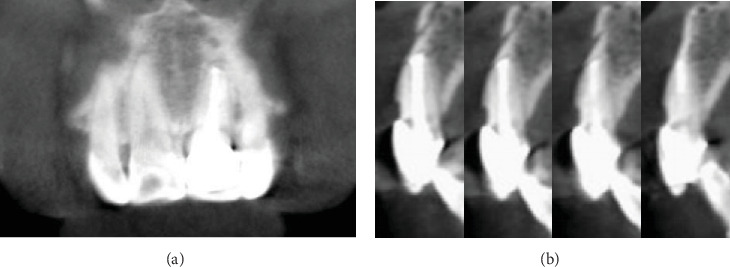
CBCT radiographic evaluation at the 12-month follow-up. (a) Coronal and (b) sagittal images showed the resolution of the low-density lesion at the apex.

To avoid any interference with periodontal health, excess cement was meticulously removed, and root planing was carefully performed. After that, the mucoperiosteal flap was sutured using 4-0 Dafilon nonabsorbable suture material (Dafilon, B. Braun, Melsungen, Germany) (Figure 2c). Postoperative instructions included avoiding hard foods on the affected tooth, maintaining optimal oral hygiene, and scheduling follow-up appointments at 1 week, 1 month, and quarterly intervals. At the 12-month follow-up, the tooth fragment showed a proper adaptation as well as good periodontal health and no evidence of root and bone resorption (Figure 3).

**Figure 4:**
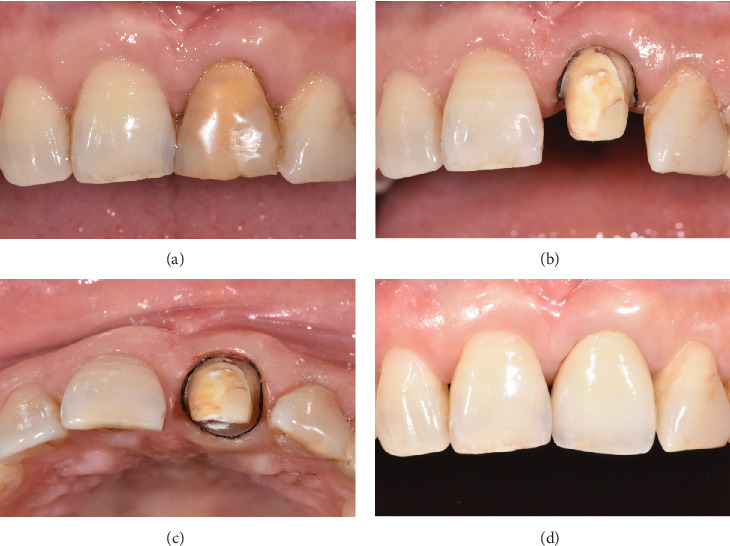
Fabrication of the final restoration. (a) Intraoral condition before tooth preparation. (b, c) Tooth preparation. (d) Placement of the final restoration.

After confirming the health of the periodontium at the 12-month follow-up, the fractured tooth was prepared for final restoration. A final all-ceramic crown was fabricated based on the patient's individual shade selection, ensuring esthetic integration. Then, the crown was tried on, adjusted for occlusion, and cemented with 3 M resin cement (RelyX Ultimate; 3M ESPE, St. Paul, United States) (Figure 4). The patient was instructed to avoid masticating hard or sticky foods on the restored tooth, maintain meticulous oral hygiene, and attend follow-up appointments every 6 months to monitor the long-term stability and health of the restoration.

### 2.5. Treatment Results

**Figure 5:**
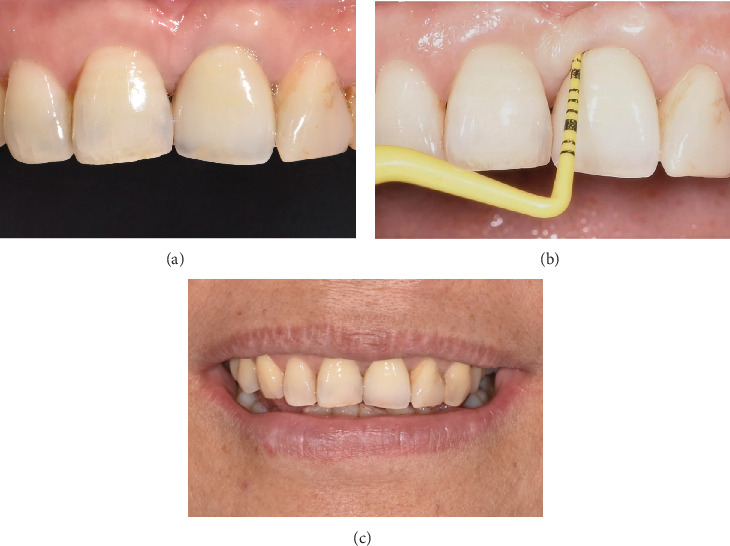
55-month follow-up showed healthy periodontium and satisfactory esthetics. (a) Gingival margins were even, with no papillary recession. (b) No bleeding on probing, with probing depths of 2–3 mm. (c) The patient was pleased with the restoration.

**Figure 6:**
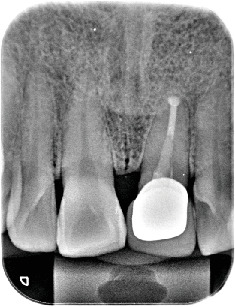
Periapical radiographic evaluation at 55-month follow-up. The radiographs revealed consistent bone density at the apex with adjacent teeth, continuous cortical bone lines on the mesial and distal alveolar crests, and no abnormalities in the periodontal ligament space and no signs of root resorption.

Regular follow-ups were conducted at 6-month intervals between the 12-month and 55-month assessments. During these visits, clinical and radiographic evaluations were performed to monitor the stability of the restoration, periodontal health, and healing of the periapical area. No complications, such as microleakage, debonding, or periodontal issues, were observed during this period. At the 55-month follow-up, the margin of the all-ceramic crown remained well-sealed, the periodontal tissues were healthy, and the color and texture of the gingiva were satisfactory, and the probing depths were less than 3 mm on each side of the labial and palatal sides; bleeding on probing was negative (Figure 5). Radiography revealed no resorption of the alveolar bone and root and complete healing of the periapical cystic area (Figure 6). The patient expressed satisfaction with both the esthetics and function of the restored tooth.

To optimize scientific validity, a lifelong follow-up protocol has been instituted, mandating biannual clinical and radiographic assessments. Evaluations encompass periodontal indices (probing depth and bleeding on probing), alveolar crest resorption, prosthetic integrity, secondary caries, and occlusal relationships. Emphasis is placed on standardized oral hygiene protocols, including modified Bass technique and precision use of interdental cleaning aids (e.g., interdental brushes and floss) to ensure therapeutic longevity.

## 3. Discussion

Crown-root fractures in the esthetic zone present significant challenges due to their potential impact on both function and appearance. As such, immediate treatment is crucial to prevent further complications.

There are many clinical treatment options for crown-root fractures, such as crown lengthening, restoration after orthodontic extrusion, or immediate implant placement after extraction [3, 6]. However, due to the patient's history of apical cyst resection and insufficient bone volume in the apical region of the tooth, immediate implantation could not achieve primary stability [15]. Crown lengthening surgery may lead to gingival margin discrepancy in the central incisors [16]. Moreover, orthodontic extrusion is often limited by its time-consuming nature [17].

Given the patient's strong desire to preserve the natural tooth and the need for long-term monitoring to assess the healing progress of the periapical region following the completion of root canal treatment, crown-root fragment reattachment was selected as the optimal treatment option in this case. This approach allows for the preservation of the natural tooth while avoiding the risks associated with other invasive procedures. The success of this technique depends on factors such as the size of the fractured segment, periodontal involvement, biological width violation, duration of the fracture, contamination, and the material used for bonding [18]. In this case, the patient presented promptly with an intact fractured fragment, where only the mesial fracture end extended about 1 mm below the alveolar crest, alongside the remaining root's length and stability, which provided favorable conditions for successful reattachment. Patil et al. reported that in situations with minimal biologic width invasion, the organism can restore the biologic width by itself, provided that the dental plaque is properly controlled [19]. A systematic review confirmed that even teeth with crown-root fractures whose biological width was violated can achieve favorable clinical outcomes after fragment reattachment [20]. Nonetheless, achieving a dry operative field is critical for proper sealing and preventing microleakage, which otherwise can damage biological width [21]. Therefore, in the process of reattachment, one of the most important steps is to achieve optimal adhesion after strict isolation of moisture and contaminants, thereby creating a healthy periodontal environment conducive to the reconstruction of periodontal homeostasis. This, in turn, allows for the re-establishment of a suitable biological width, ultimately achieving the purpose of preserving the natural tooth as much as possible.

Consequently, we selected crown reattachment combined with flap surgery and a series of measures to isolate contaminants. The documents revealed that local anesthetics containing epinephrine and gingival hemostatic gel containing ferrous sulfate were applied to effectively reduce bleeding and the amount of gingival crevicular fluid in the surgical area [12, 22]. Following the elevation of a full-thickness labial mucoperiosteal flap, dentin surface pretreatment can be performed to eliminate contaminants, thereby enhancing dentin bond strength [23]. The residual cement was carefully removed under direct vision so as to avoid the occurrence of periodontal inflammation. However, flap elevation may carry a risk of both papilla recession and mid-facial recession due to the potential disruption of the vascular supply and soft tissue integrity during surgical manipulation [24]. In this case, following the M-MIST principle, meticulous flap design, precise incision placement, and careful tissue elevation were employed to minimize biological width violation and preserve periodontal health.

Notably, the Super-Bond adhesive system, a self-curing dental adhesive resin material, differs significantly from traditional adhesives. In fact, moisture or blood contamination can reduce the adhesive performance of conventional or hydrophilic adhesives [25, 26]. Conversely, Super-Bond adhesive is not affected by little blood and moisture contamination because its polymerization is greatly enhanced when tributylborane, the catalyst, is exposed to moisture and air [27]. The formation of a hybrid layer (resin-impregnated layer) in both enamel and dentin allows excellent bonding and sealing of tooth structures (enamel and dentin) in oral environments where complete dryness is difficult to achieve. Therefore, the Super-Bond adhesive system can be effectively used to reattach fractured fragments. Moreover, studies have demonstrated that the Super-Bond adhesive system exhibits low cytotoxicity and excellent biocompatibility with periodontal tissues, making it suitable for long-term use in the oral cavity [9]. These properties contribute to maintaining periodontal health and reducing the risk of adverse tissue reactions.

Studies demonstrate that M-MIST procedures achieve significant probing depth reduction, clinical attachment level gain, and minimal recession at 1-year follow-up compared to baseline [28]. Therefore, in this case, an all-ceramic crown was permanently restored 12 months after flap surgery, following the confirmation of periodontal health, to restore the esthetic effect of the discolored tooth. The case has been followed for 55 months with favorable clinical outcomes and excellent patient satisfaction.

## 4. Conclusion

This clinical case demonstrated that in an oral environment where complete moisture control is impossible, utilizing Super-Bond for immediate crown-root fragment reattachment under a small periodontal flap is suitable for achieving favorable esthetic restoration outcomes and promoting periodontal health. Limitations include that this method may not be suitable for teeth with severely damaged biological width.

## Figures and Tables

**Table 1:** Phased therapeutic protocol based on conservative and minimally invasive principles for crown-root fragment reattachment.

**Phase I: Immediate reattachment**	**Phase II: Definitive restoration**	**Phase III: Long-term monitoring**
Goal: Reattach fractured crown-root fragment with adhesive techniques.	Goal: Place full-ceramic crown for long-term protection and esthetics.	Goal: Monitor periapical healing and restoration stability.
Key procedures: -Moisture control and precise fragment alignment. -Reinforcement with fiber post for structural support. -Adhesive reattachment using Super-Bond system.	Key procedures: -Tooth preparation and impression for ceramic crown. -Cementation of ceramic crown with resin luting agent. -Occlusal adjustment and polishing.	Key evaluations: -Clinical assessment of periodontal health, secondary caries, crown integrity, and occlusal contact. -Radiographic evaluation of periapical status and alveolar bone resorption.
Follow-up schedule: 1 week, 1 month, 3 months, 6 months, 9 months, and 12 month.	Follow-up schedule: Every 6 months.	Follow-up schedule: Every 6 months, lifelong maintenance recommended.

## Data Availability

The data that support the findings of this study are available from the corresponding author upon reasonable request.
